# The *Saccharomyces cerevisiae* Cdk8 Mediator Represses *AQY1* Transcription by Inhibiting Set1p-Dependent Histone Methylation

**DOI:** 10.1534/g3.117.039586

**Published:** 2017-01-30

**Authors:** Michael J. Law, Michael A. Finger

**Affiliations:** Department of Molecular Biology, Rowan University School of Osteopathic Medicine, Stratford, New Jersey 08084

**Keywords:** histone modifications, mediator, transcription factors

## Abstract

In the budding yeast *Saccharomyces cerevisiae*, nutrient depletion induces massive transcriptional reprogramming that relies upon communication between transcription factors, post-translational histone modifications, and the RNA polymerase II holoenzyme complex. Histone H3Lys4 methylation (H3Lys4 me), regulated by the Set1p-containing COMPASS methyltransferase complex and Jhd2p demethylase, is one of the most well-studied histone modifications. We previously demonstrated that the RNA polymerase II mediator components cyclin C-Cdk8p inhibit locus-specific H3Lys4 3me independently of Jhd2p. Here, we identify loci subject to cyclin C- and Jhd2p-dependent histone H3Lys4 3me inhibition using chromatin immunoprecipitation (ChIP)-seq. We further characterized the independent and combined roles of cyclin C and Jhd2p in controlling H3Lys4 3me and transcription in response to fermentable and nonfermentable carbon at multiple loci. These experiments suggest that H3Lys4 3me alone is insufficient to induce transcription. Interestingly, we identified an unexpected role for cyclin C-Cdk8p in repressing *AQY1* transcription, an aquaporin whose expression is normally induced during nutrient deprivation. These experiments, combined with previous work in other labs, support a two-step model in which cyclin C-Cdk8p mediate *AQY1* transcriptional repression by stimulating transcription factor proteolysis and preventing Set1p recruitment to the *AQY1* locus.

Complex signaling networks are in place that allow cells to sense stressful conditions and transduce signals to transcription factors and chromatin regulatory complexes, resulting in dramatic transcriptional reprogramming. The budding yeast *Saccharomyces cerevisiae* is a well-defined experimental system to understand complex transcriptional responses to nutritional information. In the presence of fermentable carbons such as glucose, yeast express growth genes including ribosomal proteins, rRNA processing genes, and glycolytic enzymes, permitting increased biomass and rapid cellular divisions (DeRisi *et al.* 1997). In the absence of fermentable carbon, yeast will induce the expression of genes involved in gluconeogenesis and respiration, both of which are required to maintain cell growth (Barnett and Entian 2005). More severe nutrient deprivation can lead diploid yeast cells to differentiate to form haploid gametes via meiosis or to enter nutritional foraging via pseudohyphal growth (Esposito and Klapholz 1981; Gimeno *et al.* 1992). Therefore, the severity of the nutritional stress dictates important cell fate decisions in yeast. Understanding the transcriptional mechanisms that regulate these morphological switches has been the focus of intensive investigations from multiple laboratories [reviewed in Schuller (2003) and Turcotte *et al.* (2010)].

Post-translational histone modifications, including acetylation, phosphorylation, ubiquitination, and methylation, function in all aspects of chromatin biology and together act to control transcriptional activation and repression (Rando and Winston 2012). H3Lys4 me, regulated by the opposing activities of the Set1p-containing COMPASS methyltransferase complex and the Jhd2 demethylase, is one of the most well-studied histone modifications (Roguev *et al.* 2001; Boa *et al.* 2003; liang *et al.* 2007). H3Lys4 can be methylated up to three times, with each methylation level resulting in a different cellular interpretation (berger 2007). While early studies suggested that H3Lys4 3me stimulates transcription, recent work has suggested more controversial roles for H3Lys4 me in controlling both transcriptional activation and repression (Kim and Buratowski 2009; Margaritis *et al.* 2012; D’Urso *et al.* 2016). Genetic and biochemical studies have demonstrated that specific members of the COMPASS methyltransferase complex are required to catalyze precise H3Lys4 me levels. These investigations suggest that one way in which cells may control locus-specific H3Lys4 me levels and transcription is by remodeling the COMPASS complex. Identifying regulators of COMPASS complex dynamics has been complicated by the fact that many studies have been performed *in vitro* or using steady-state growth conditions.

Recent studies have indicated that COMPASS interacts genetically and biochemically with the CDK8 mediator complex to regulate cellular response to nutrient deprivation. The CDK8 complex, composed of Ssn8p, Ssn3p, Srb8p, and Ssn2p (herein referred to as cyclin C, Cdk8p, Med12p, and Med13p, respectively), is a locus-specific transcriptional regulator of stress responsive and developmental genes in yeast (Kuchin *et al.* 1995; Cooper *et al.* 1997; Bourbon *et al.* 2004; van de Peppel *et al.* 2005). The cyclin C-Cdk8p kinase complex regulates transcription by phosphorylating a wide range of substrates including components of the RNA pol II holoenzyme and transcription factors (Liao *et al.* 1995; Hirst *et al.* 1999; Chi *et al.* 2001; Zaman *et al.* 2001; Nelson *et al.* 2003; Raithatha *et al.* 2012) . Recent work suggests that in addition to these substrates, cyclin C-Cdk8p may regulate COMPASS as cells respond to nutrient depletion . For example, Cdk8p controls locus-specific COMPASS remodeling and transcriptional memory establishment in response to inositol starvation (D’Urso *et al.* 2016). In addition, work from our laboratory demonstrated that cyclin C-Cdk8p and Jhd2p inhibit pseudohyphal growth while cells are cultured in rich fermentative conditions (Law and Ciccaglione 2015). Work from this study also revealed that cyclin C-Cdk8p inhibit locus-specific H3Lys4 3me independently of Jhd2p, suggesting that cyclin C-Cdk8p-mediated transcriptional controls may act in part through histone H3Lys4 methylation (Law and Ciccaglione 2015).

Here, we used ChIP-seq on yeast cultured in nonfermentable carbon to identify loci displaying aberrant H3Lys4 3me patterns in *cnc1*Δ*jhd2*Δ mutants. We further characterized how carbon source controls both H3Lys4 3me and RNA expression and found that *CNC1- JHD2*-dependent H3Lys4 3me is sensitive to carbon source at some loci, but not others. Interestingly, we discovered that cyclin C-Cdk8p inhibit *AQY1* mRNA expression, a gene that is induced during pseudohyphal growth and meiosis, by preventing Set1p binding to the *AQY1* promoter. Together, our results indicate that cyclin C-Cdk8p restrict H3Lys4 me at stress responsive and metabolic genes, and that inactivating this kinase complex is an important step during cellular response to adverse growth conditions.

## Materials and Methods

### Yeast strains and growth conditions

Yeast strains are listed in Table 1 and are derived from the SK1 genetic background. Yeast were cultured in YEPD (1% yeast extract, 2% peptone, and 2% dextrose) or YEPA (1% yeast extract, 2% peptone, and 1% potassium acetate) to midlogarithmic phase as determined by hemocytometric quantification.

**Table 1:** Yeast strains used in this study

Strain	Genotype*^a^*	Source
RSY883	MATa/MATα lys2 trp1::hisG ura3 LYS2::ho∆	Strich *et al.* (2004)
MLY2	MATa/MATα lys2 trp1::hisG ura3 LYS2::ho∆ cnc1::TRP1 jhd2::KanMX	Law and Ciccaglione (2015)
MLY3	MATa/MATα lys2 trp1::hisG ura3 LYS2::ho∆ jhd2::KanMx	Law and Ciccaglione (2015)
MLY4	MATa/MATα lys2 trp1::hisG ura3 LYS2::ho∆ cnc1::TRP1	Law and Ciccaglione (2015)
MLY19	MATa/MATα lys2 trp1::hisG ura3 LYS2::ho∆ SET1-9MYC::TRP1 cdk8::KanMX	This study
MLY20	MATa/MATα lys2 trp1::hisG ura3 LYS2::ho∆ SET1-9MYC::TRP1	This study

a All strains are derived from the SK1 genetic background and are isogenic to RSY883; genotypes are homozygous diploids.

### RT-qPCR

Total nucleic acids were prepared from 20 ml midlogarithmic cultures. Approximately 500 ng of total nucleic acid preparations were then treated with DNase I (New England Biolabs), followed by reverse transcription using Protoscript II reverse transcriptase (New England Biolabs) in oligo-dT primed reactions according to the manufacturer’s instructions. Subsequent qPCR reactions were prepared using the Power SYBR Master mix (Applied Biosystems) containing primers listed in Supplemental Material, Table S1. All C_T_ values were normalized first to *ACT1*, then to wild-type values (ΔΔC_T_). Values reported are the average of three or more independent biological replicates; error bars represent the SDs.

### ChIP

ChIP was performed essentially as described previously (Law and Ciccaglione 2015) with the following modifications. First, 50 or 100 ml of midlogarithmic dextrose or acetate cultures, respectively, were cross-linked with 1% formaldehyde (15 min at room temperature) followed by quenching of cross-linked protein/DNA complexes with 140 mM glycine for 5 min. Cross-linked cells were then spheroplasted, washed extensively, and sonicated using a Bioruptor UCD-200 (Diagenode) to generate fragments ∼300–750 nt in length. For ChIP-seq experiments, immunoprecipitations were performed on 50 μg of chromatin solution that were first precleared with protein G Dynabeads (LifeTech 10004D) using antibodies directed toward trimethylated H3 Lys4 (Abcam, ab8580) or histone H3 C-terminal domain (CTD) (Abcam, ab1791). For ChIP-qPCR experiments, immunoprecipitations were performed on 50 μg of chromatin solution that were precleared with protein G agarose (Sigma P-7700) using antibodies directed toward mono-, di-, or trimethylated H3 Lys4 (Cell Signaling Technologies cat no. 5326 1me, 9725 2me, and 9727 3me) or histone H3 CTD (Abcam ab1791). ChIP-qPCR measuring myc-Set1p occupancy was performed by incubating 500 μg of chromatin solution harboring chromosomally integrated 9-myc-Set1p or untagged wild yeast strains with 20 μl myc-conjugated agarose overnight at 4° with gentle rocking.

Immune complexes were collected using Dynabeads (ChIP-seq) or protein G agarose (ChIP-qPCR) and washed sequentially with TSE-150 and -500, LiCl/detergent, and TE. Beads were then treated with RNase A and elutions were performed by incubation in 1% SDS/TE at 65° for 15 min. Cross-links were reversed by incubating overnight at 65° followed by proteinase K (Roche) treatment according to the manufacturer’s instructions. DNA was purified using QIAquick PCR column purification (QIAGEN, cat. no. 28106) according to the manufacturer’s instructions. For qPCR, % input of each IP was calculated using a standard curve for each primer pair. ChIP-seq was performed on biological replicates; ChIP-qPCR was performed on three or more independent biological repeats with results reporting averages with error bars indicating SDs.

### Library preparation and next-generation sequencing

ChIP-seq libraries were prepared on immunoprecipitated eluates by first performing end repair (New England Biolabs; T4 DNA polymerase M0203, T4 polynucleotide kinase M0201, and Klenow DNA polymerase M0210) and adding A bases (New England Biolabs Klenow fragment M0212) prior to adapter ligation (New England Biolabs, Quick T4 DNA ligase M2200). Adapter-ligated immunoprecipitated DNA was then purified using Agencourt AMPure XP beads (Beckman Coulter A63880). Size selection was performed by extracting bands from a 2% agarose gel (Bio-Rad, Certified Low Range Ultra agarose 161-3106) whose apparent molecular weight was ∼225 bp. Gel slices were purified using a QIAquick gel extraction kit according to the manufacturer’s instructions (QIAGEN cat. no. 28706). Materials were enriched by low cycle PCR using oligonucleotides that contain barcodes for multiplex next-generation sequencing (18 cycles, Phusion, Life Technologies F530). Library validation and quantification was performed using Agilent DNA-1000 kit on Bioanalyzer instrumentation.

Next-generation sequencing was performed at the University of Pennsylvania’s functional genomics core using an Illumina HiSequation 2000 instrument to generate 50 bp single end reads.

### Computational analyses

Quality control of fastq files was performed using FastQC (Andrews 2010). FastQC did not identify any flags in the quality of the sequenced libraries and therefore no further preprocessing was performed. Reads were aligned to the SK1 reference genome using BWA (bwa aln; bwa samse) with the default parameters (Li and Durbin 2009; Li 2013). Peaks were called with histone H3 immunoprecipitations serving as the input control using MACS2 with the following parameters: “callpeak -g 1.2E7 -p 0.01–nomodel–extsize147–tolarge” (Zhang *et al.* 2008). Differential peaks were identified using DiffBind with the default edgeR parameters (v1.16.3; Stark and Brown 2011; Ross-Innes *et al.* 2012); annotations were performed using ChIPPeakAnno (v3.8.0; Zhu *et al.* 2010; Zhu 2013) and HOMER (v 4.8; Heinz *et al.* 2010).

### Data availability

Strains are available upon request. Raw ChIP-seq data (.fastq) and mapped reads used for analyses reported in this manuscript are available on GEO, accession number GSE93641. 

## Results

### Cyclin C and Jhd2p inhibit locus-specific histone H3Lys4 trimethylation

**Figure 1:**
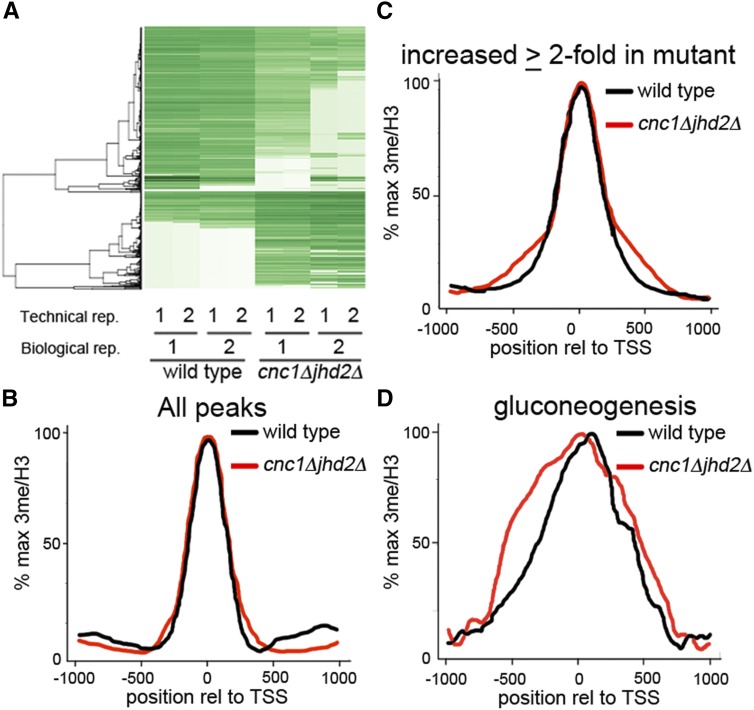
Differential peak identification and H3Lys4 3me distribution relative to TSS. (A) Hierarchical clustering of differential H3Lys4 3me peaks as determined by DiffBind. After peak calling, wild type and *cnc1*Δ*jhd2*Δ mutants were analyzed for differential peaks. For each genotype, two biological replicates with two technical sequencing replicates were performed. Green = strong peak, white = no peak. (B–D) Distribution of H3Lys4 3me relative to histone H3 across the TSS for (B) all peaks (C) peaks that are ≥twofold elevated in *cnc1*Δ*jhd2*Δ mutants, and (D) peaks for genes involved in gluconeogenesis. H3Lys4 3me, histone H3Lys4 trimethylation; TSS, transcriptional start sites.

Our previous work indicated that cyclin C and Jhd2p inhibit histone H3Lys4 3me at the *FLO11* promoter (Law and Ciccaglione 2015). To identify loci that display similar methylation patterns, we performed ChIP-seq. Immunoprecipitations using antibodies that recognize total histone H3 CTD or histone H3Lys4 3me were performed on chromatin solutions from biological replicates of wild type or *cnc1*Δ*jhd2*Δ yeast mutants cultured in medium lacking fermentable carbon. We selected this growth condition because the majority of reported H3Lys4 me analyses have been performed in dextrose media. Following next-generation sequencing, we identified differential histone H3Lys4 3me patterns between wild-type and mutant samples (Figure S1). To account for ChIP-seq biases that could result from uneven genomic coverage, we opted to use histone H3 CTD immunoprecipitations as our background control instead of “input” samples (Flensburg *et al.* 2014). Read quality was assessed on all fastq files and mapping efficiency was determined prior to peak calling (Figure S1 and Figure S2). Differential peak analyses identified 591 loci displaying a ≥twofold elevation in H3Lys4 3me levels in mutant cells relative to wild type (Figure 1A, Table S2, and Table S3). Conversely, we detected 465 loci with elevated H3Lys4 3me levels in wild type relative to mutant yeast; however, these peaks displayed reduced amplitudes compared to those observed in mutant yeast (394 loci increased ≥eightfold in mutants v. 112 in wild type; Table S2, Table S3, and Table S4). The remainder of this manuscript will therefore focus on those loci displaying elevated H3Lys4 3me in *cnc1*Δ*jhd2*Δ mutants.

We next validated the identified H3Lys4 3me peaks by manually inspecting mapped reads from top hits including *TDH1*, *INO1*, *ENO1*, and *AQY1* using the Integrative Genomics Viewer (IGV; Robinson *et al.* 2011; Thorvaldsdottir *et al.* 2013; Figure S3A). Peaks were further confirmed by performing ChIP-qPCR using antibodies that recognize either histone H3Lys4 3me or total histone H3 on wild type and *cnc1*Δ*jhd2*Δ mutants cultured in nonfermentable carbon. These experiments confirmed that H3Lys4 3me levels are reproducibly elevated in *cnc1*Δ*jhd2*Δ mutants at the *TDH1*, *INO1*, *ENO1*, and *AQY1* loci (Figure S3B). Together, these data indicate that cyclin C and Jhd2p inhibit locus-specific methylation for yeast cultured in nonfermentable growth conditions.

### Cyclin C and Jhd2p control both the magnitude and distribution of H3Lys4 3me at stress responsive genes

We were next interested in identifying which types of genes harbor hypermethylated histone H3Lys4 in *cnc1*Δ*jhd2*Δ mutants. Gene Ontology (GO) analyses of the affected loci revealed an overrepresentation of genes involved in carbon metabolism, osmotic stress, and negative regulators of pseudohyphal growth (Table S5). This is in agreement with previous reports indicating that cyclin C-Cdk8p repress the transcription of genes involved in these processes and with the constitutive pseudohyphal phenotype observed in *cnc1*Δ*jhd2*Δ yeast mutants (Law and Ciccaglione 2015).

We wished to determine if, in addition to inhibiting the magnitude of H3Lys4 3me at target genes, cyclin C and Jhd2p impact H3Lys4 3me distribution. To do this, we performed analyses of H3Lys4 3me distribution relative to transcriptional start sites (TSS) on a genome-wide basis by evaluating all peaks and those peaks that are ≥twofold elevated in *cnc1*Δ*jhd2*Δ yeast mutants (Figure 1, B and C). These data indicate that global H3Lys4 3me distribution is similar in both wild type and *cnc1*Δ*jhd2*Δ yeast mutants, which is consistent with our previous observations that support a locus-specific control mechanism (Law and Ciccaglione 2015).

Since genes involved in the same biological process are likely to have overlapping regulatory mechanisms, we hypothesized that any differences in H3Lys4 3me distribution may be restricted to those genes that control a singular process. To test this hypothesis, we generated H3Lys4 3me TSS curves for genes involved in one of our top hits from GO analyses, gluconeogenesis (Table S5). Surprisingly, we observed a broad 5′ shift in H3Lys4 3me distribution gluconeogenic genes for *cnc1*Δ*jhd2*Δ yeast mutants (Figure 1D). Similar analyses performed for carbon metabolic genes and negative regulators of pseudohyphal growth revealed that *cnc1*Δ*jhd2*Δ yeast mutants display aberrant H3Lys4 3me patterns relative to wild-type cells (Figure S4). Together, these analyses indicate that cyclin C and Jhd2p regulate the locus-specific magnitude and distribution of H3Lys4 3me.

### Locus-specific methylation patterns are sensitive to growth conditions

We were interested in determining the individual and combined contribution of cyclin C and Jhd2p to locus-specific H3Lys4 3me and what role, if any, carbon source plays in this process. To do this, we performed ChIP-qPCR experiments in wild type and single or double *cnc1*Δ *jhd2*Δ mutant yeast cultured in either rich, fermentative, or nonfermentative growth conditions. Immunoprecipitations were directed toward histone H3Lys4 3me or total histone H3, and qPCR reactions were directed toward genes involved in carbohydrate metabolism, *TDH1*, *INO1*, and *ENO1*, or an aquaporin that is important for stress tolerance, *AQY1*.

**Figure 2:**
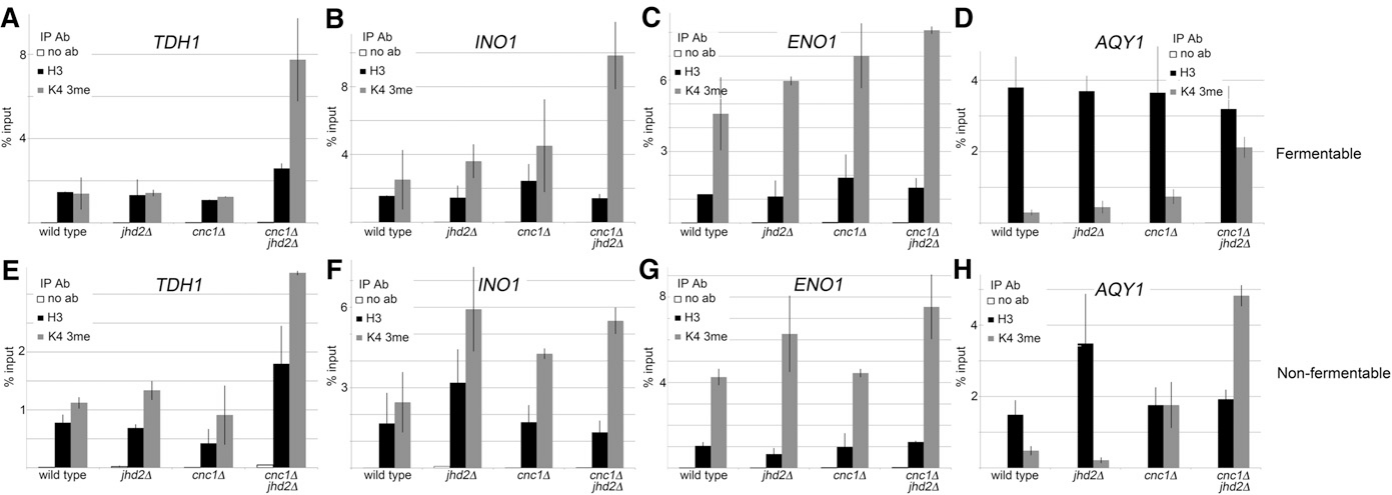
ChIP-qPCR measuring H3Lys4 3me and histone H3 abundance at differentially bound loci for yeast cultured in fermentable and nonfermentable carbon. ChIP-qPCR was performed for wild type, *jhd2*Δ, *cnc1*Δ, and *cnc1*Δ*jhd2*Δ mutants cultured to midlogarithmic phase in either fermentable (top) or nonfermentable (bottom) carbon. qPCR reactions targeted loci displaying enrichment as determined by ChIP-seq; (A and E) *TDH1*, (B and F) *INO1*, (C and G) *ENO1*, and (D and H) *AQY1*. ChIP signal was calculated by normalization to standard curves for each individual primer pair and then determining the percent input of each immunoprecipitation. Histograms represent the average percent input from three independent biological replicates, error bars are SD. Ab, antibody; ChIP, chromatin immunoprecipitation; ChIP-seq, chromatin immunoprecipitation sequencing; H3Lys4 3me, histone H3Lys4 trimethylation; IP, immunoprecipitation; qPCR, quantitative polymerase chain reaction.

First, we observed minimal impact on H3Lys4 3me levels in either *cnc1*Δ or *jhd2*Δ single mutants at the *TDH1* locus regardless of growth condition (Figure 2, A and E). Removal of both *CNC1* and *JHD2* resulted in elevated H3Lys4 3me, suggesting that they play a redundant role in inhibiting H3Lys4 3me at this locus.

Next, we found minor increases in H3Lys4 3me levels for *cnc1*Δ and *jhd2*Δ single mutants cultured in fermentable carbon at the *INO1* locus. However, *cnc1*Δ*jhd2*Δ yeast mutants displayed dramatic H3Lys4 3me increases in this growth condition (Figure 2B). Interestingly, H3Lys4 3me patterns changed for yeast cultured in nonfermentable carbon with *jhd2*Δ and *cnc1*Δ*jhd2*Δ yeast mutants displaying similar H3Lys4 3me levels (Figure 2F). This suggests that cyclin C-Cdk8p activity at the *INO1* locus may be inhibited in the absence of fermentable carbon.

Cyclin C and Jhd2p play nonredundant roles in inhibiting H3Lys4 3me levels at the *ENO1* locus while cells are cultured in fermentable carbon, as we observed additive increases in H3Lys4 3me for *cnc1*Δ*jhd2*Δ mutants relative to each single mutant (Figure 2C). Contrary to this, cells cultured in nonfermentable carbon displayed mainly *JHD2*-mediated H3Lys4 3me repression, supporting a similar regulatory model for both *INO1* and *ENO1* (Figure 2G).

Finally, we found that both cyclin C and Jhd2p inhibit H3Lys4 3me at *AQY1* during growth in fermentable carbon, since elevated H3Lys4 3me is only observed in *cnc1*Δ*jhd2*Δ mutants (Figure 2D). In contrast, cells cultured in nonfermentable carbon displayed partial *CNC1*-mediated H3Lys4 3me inhibition, which was dramatically increased in yeast lacking both *CNC1* and *JHD2* (Figure 2H). Together, these data support a model in which cyclin C and Jhd2p make different, locus-specific contributions to H3Lys4 3me, and that these contributions are sensitive to carbon source.

### H3Lys4 3me-dependent transcriptional controls are mediated by cyclin C and Jhd2p

**Figure 3:**
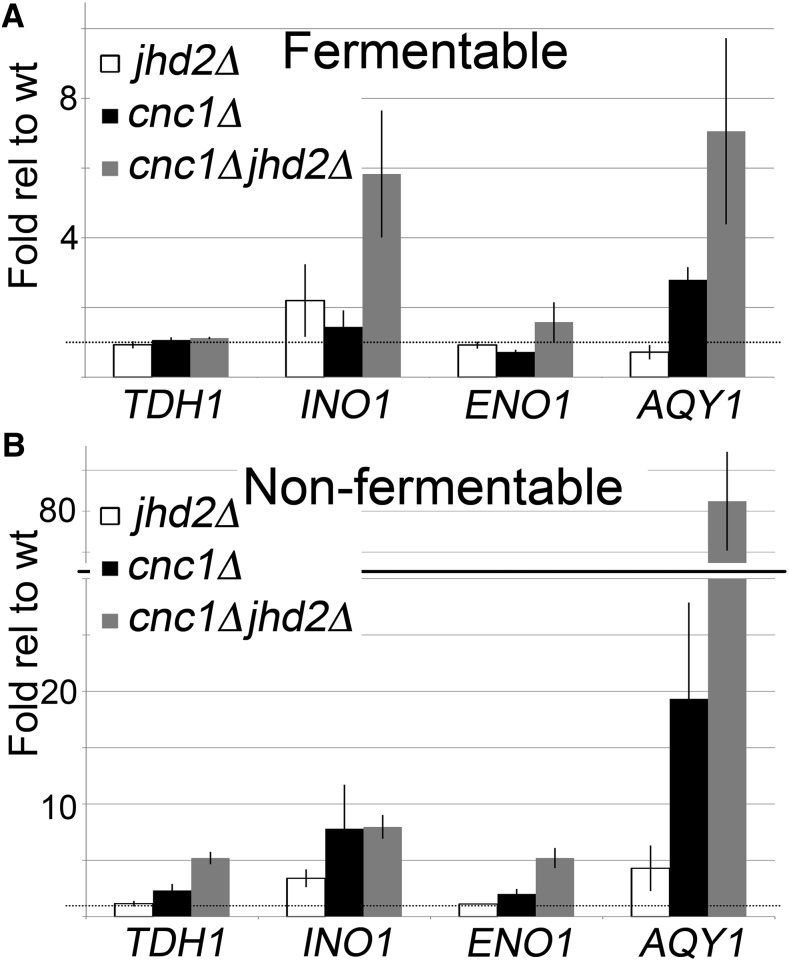
RT-qPCR measuring the expression of cyclin C and Jhd2p target genes. Reverse transcription was performed on wt, *jhd2*Δ, *cnc1*Δ, and *cnc1*Δ*jhd2*Δ yeast mutants cultured to midlogarithmic phase in either (A) fermentable or (B) nonfermentable carbon. qPCR reactions were directed toward *TDH1*, *INO1*, *ENO1*, and *AQY1* and were normalized to *ACT1*. Signal for wt was set equal to one (ΔΔC_T_). Histograms represent the average of three independent biological replicates, error bars indicate SD. RT-qPCR, quantitative reverse transcription polymerase chain reaction; wt, wild type.

Since H3Lys4 3me is often correlated with active transcription, we were next interested in determining the transcriptional impact of elevated methylation at target loci described above. RT-qPCR experiments were performed on wild type and single or double *cnc1*Δ *jhd2*Δ mutants cultured in fermentable or nonfermentable carbon sources. RNA levels for *TDH1* remain unchanged during growth in fermentable carbon in all genotypes, despite dramatically increased H3Lys4 3me levels in *cnc1*Δ*jhd2*Δ mutants in this growth condition (Figure 3A). This suggests that elevated H3Lys4 3me is not sufficient to drive transcription of this gene. In contrast, slightly elevated *TDH1* levels were observed in *cnc1*Δ*jhd2*Δ mutants cultured in nonfermentable carbon (Figure 3B), indicating that failure to transcribe this target while cells are grown in fermentable carbon may result from glucose catabolite repression. In support of this notion, *TDH1* is expressed during stationary phase when glucose is depleted, while the other enzymatic isoforms *TDH2* and *TDH3* are expressed during logarithmic growth (Delgado *et al.* 2001).

*INO1* mRNA levels are increased ∼twofold in *jhd2*Δ mutants cultured in fermentable carbon, despite little to no change in H3Lys4 3me levels in this mutant (Figure 2B and Figure 3A). Additional *INO1* derepression is observed in *cnc1*Δ*jhd2*Δ mutants (∼sixfold), indicating that cyclin C and Jhd2p play nonredundant roles in repressing *INO1* transcription. In contrast, both *cnc1*Δ and *cnc1*Δ*jhd2*Δ mutants cultured in nonfermentable carbon displayed similar levels of *INO1* derepression (Figure 3B). This suggests that *INO1* repression requires only cyclin C while cells are grown in nonfermentable carbon, in agreement with previous studies performed in other laboratories (D’Urso *et al.* 2016).

*ENO1* expression appears unaffected by cyclin C and Jhd2p while cells are grown in fermentable carbon (Figure 3A). In addition, transcription is only marginally increased in *cnc1*Δ*jhd2*Δ mutants grown in nonfermentable carbon, suggesting that multiple cyclin C and Jhd2p-independent transcriptional control mechanisms are in place for this gene (Figure 3B).

Finally, we observed a moderate ∼twofold derepression of *AQY1* transcription in *cnc1*Δ mutants while *cnc1*Δ*jhd2*Δ mutants displayed a ∼sixfold increase for cultures grown in fermentable carbon (Figure 3A). Interestingly, we observed synergistic repression of *AQY1* transcription while yeast were cultured in nonfermentable carbon; *AQY1* levels were increased ∼fourfold in *jhd2*Δ yeast mutants, ∼20-fold in *cnc1*Δ yeast mutants, and ∼80-fold in *cnc1*Δ*jhd2*Δ mutants (Figure 3B). These results suggest that cyclin C and Jhd2p contribute to *AQY1* transcriptional repression through nonoverlapping mechanisms while cells are grown in nonfermentable carbon. In addition, these data indicate that histone H3Lys4 3me levels may be a poor predictor of transcriptional activation (compare Figure 2A and Figure 3A *TDH1* to Figure 2D and Figure 3A *AQY1*), and suggest the presence of a second locus-specific signal that is required for transcriptional induction.

### Cyclin C inhibits histone H3Lys4 methylation at the AQY1 locus during fermentative growth

**Figure 4:**
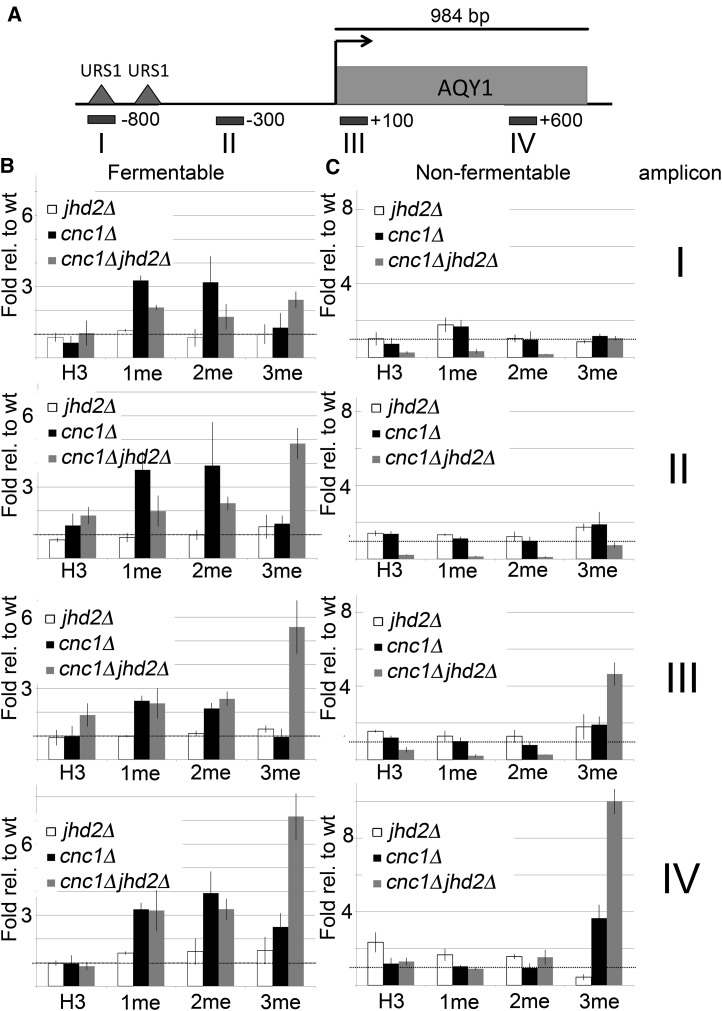
H3Lys4 methylation characterization at the *AQY1* locus (A). ChIP-qPCR was performed on wt, *jhd2*Δ, *cnc1*Δ, and *cnc1*Δ*jhd2*Δ mutants cultured in either fermentable (B) or nonfermentable (C) carbon sources. Immunoprecipitations were carried out using antibodies that recognize histone H3, or each individual H3Lys4 methylation level (1me, 2me, or 3me). To adequately map H3Lys4 methylation abundance across the *AQY1* locus, qPCR reactions were performed using four separate amplicons (I–IV). Histograms represent the average of two independent biological replicates, error bars indicate SD. ChIP, chromatin immunoprecipitation; H3Lys4, histone H3Lys4; 1me, monomethylation; 2me, dimethylation; 3me, trimethylation; qPCR, quantitative polymerase chain reaction; wt, wild type.

Since different H3Lys4 methylation levels are correlated with different transcriptional responses, we next asked which methylation levels are controlled by cyclin C and Jhd2p at the *AQY1* locus. To do this, we performed ChIP-qPCR in wild type or yeast mutants lacking *CNC1* and *JHD2* in isolation or in tandem. Yeast were cultured in either fermentable or nonfermentable carbon and immunoprecipitations were directed toward histone H3 or histone H3Lys4 1me, 2me, or 3me. To allow for efficient mapping of each H3Lys4 methylation level, qPCR reactions were directed toward four regions of the *AQY1* locus (Figure 4A). These experiments found that cyclin C represses H3Lys4 1me and 2me during vegetative growth in fermentable carbon across the entire *AQY1* locus (Figure 4B). We observed that increased H3Lys4 3me levels were restricted to *cnc1*Δ*jhd2*Δ mutants and were most pronounced at regions closest to the *AQY1* ORF (Figure 4B). This is consistent with the increased *AQY1* transcript levels in *cnc1*Δ*jhd2*Δ mutants cultured in fermentable carbon and with the observation that Jhd2p overexpression reduces global H3Lys4 3me levels, but has more minor impacts on 1me and 2me levels (Ramakrishnan *et al.* 2016).

Unlike the observations during growth in fermentable carbon, cyclin C and Jhd2p have no impact on H3Lys4 1me and 2me during growth in nonfermentable carbon (Figure 4C). This suggests that cyclin C and Jhd2p may be inactivated in this growth condition, and is supported by the observation that H3Lys4 1me and 2me are elevated in wild-type cells cultured in nonfermentable carbon relative to their fermentable levels. Close examination indicates that *cnc1*Δ*jhd2*Δ mutants display reduced histone H3 signal relative to wild-type yeast in amplicons I–III, suggesting that the chromatin in this region may be in a more open conformation. Histone H3Lys4 3me is increased in both *cnc1*Δ and *cnc1*Δ*jhd2*Δ mutants at the 3′-end of the ORF, which is consistent with the increased transcript levels observed in these mutants (Figure 4C, amplicon IV). These data indicate that cyclin C-mediated repression of histone H3Lys4 methylation levels at the *AQY1* locus is restricted to rich, fermentative growth conditions.

### Set1p occupancy at the AQY1 locus is inhibited by Cdk8p

**Figure 5:**
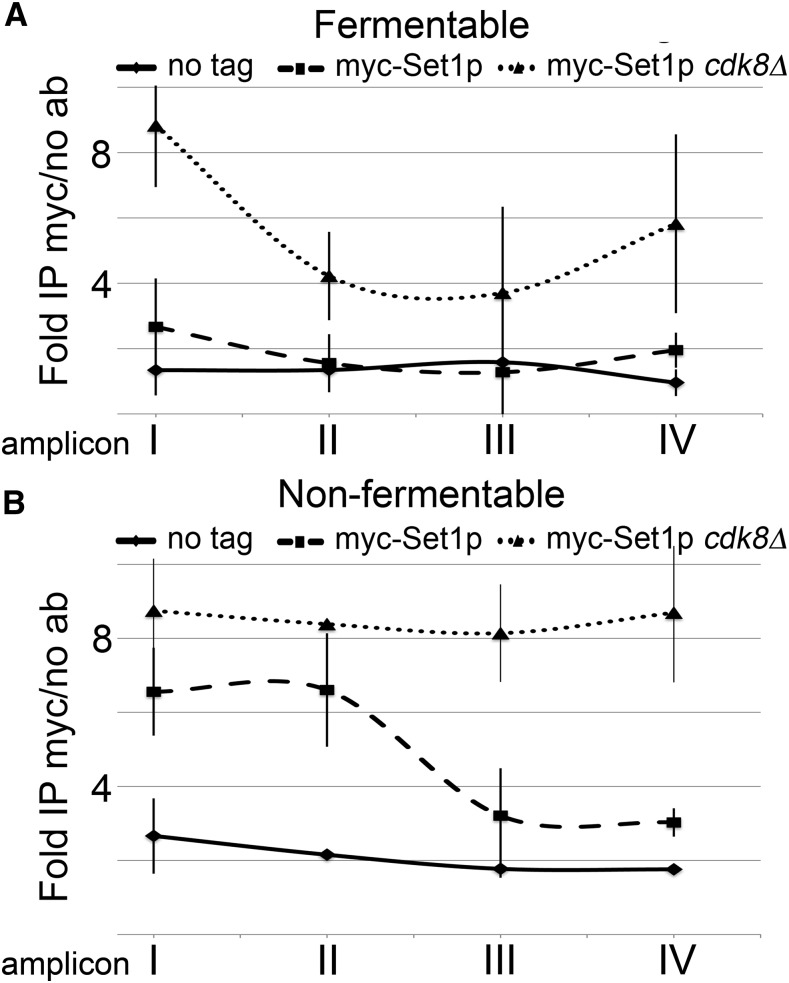
*CDK8*-dependent Set1p occupancy at the *AQY1* locus. ChIP-qPCR was performed on wild type or *cdk8*Δ mutant yeast harboring a chromosomally integrated 9-myc tag at the *SET1* locus. Yeast were cultured to midlogarithmic phase in either (A) fermentable or (B) nonfermentable carbon sources. Immunoprecipitations were performed using myc-conjugated agarose beads with wild-type untagged yeast serving as a negative control. qPCR reactions were directed at the amplicons I–IV that span the *AQY1* locus as described in Figure 4. Ab, antibody; ChIP, chromatin immunoprecipitation; IP, immunoprecipitation; qPCR, quantitative polymerase chain reaction.

Since *CNC1* encodes a cyclin that interacts with and activates Cdk8p, we were interested in understanding if H3Lys4 methylation regulation is dependent upon cyclin C-Cdk8p kinase activity. Our data are consistent with a model in which cyclin C-Cdk8p inhibit H3Lys4 methylation; therefore, we wished to determine if this inhibition is due to control of Set1p catalysis or promoter binding. To discriminate between these possibilities, we performed ChIP-qPCR on yeast harboring a chromosomally integrated myc epitope-tagged Set1p using antibodies that recognize the myc epitope. These experiments were performed in wild type or *cdk8*Δ yeast mutants grown in fermentable or nonfermentable carbon sources with untagged yeast serving as a negative control. Set1p occupancy was mapped across the entire *AQY1* locus using the amplicons described in Figure 4A. We found *CDK8*-dependent inhibition of Set1p occupancy at the *AQY1* locus during growth in fermentable carbon (Figure 5A). In contrast, cells grown in nonfermentable carbon display similar levels of Set1p in the *AQY1* promoter region in both wild type and *cdk8*Δ mutants, and *CDK8*-mediated Set1p inhibition is restricted to the *AQY1* ORF (Figure 5B). These results are consistent with our H3Lys4 methylation characterization of the *AQY1* locus, in which we observed increased H3Lys4 methylation in *cnc1*Δ mutants grown in fermentable carbon but that these increases are reduced as cells are shifted to nonfermentable carbon (Figure 4; compare left column to right column). These data support a model in which cyclin C-Cdk8p restrict locus-specific H3Lys4 methylation by inhibiting Set1p promoter binding, but not catalysis.

## Discussion

Studies in yeast and mammalian cells have identified the cyclin C-Cdk8p complex as a critical component of gene expression in response to extracellular signals during stress response, differentiation, and development [reviewed in Nemet *et al.* (2014)]. Work in yeast demonstrated that cyclin C-Cdk8p repress the transcription of many genes involved in diauxic shift and metabolism (Holstege *et al.* 1998; van de Peppel *et al.* 2005). Many molecular targets have been identified as important mediators of locus-specific transcriptional controls by cyclin C-Cdk8p. First, *in vitro* and *in vivo* studies indicate that the CDK8 complex prevents association between RNA pol II and the core mediator, thus inhibiting transcription (Naar *et al.* 2002; Knuesel *et al.* 2009; Ebmeier and Taatjes 2010). Second, the cyclin C-Cdk8p kinase phosphorylates transcriptional activators of stress responsive genes, including Ste12p, Phd1p, Msn2p, and Gcn4p, to stimulate their proteolysis (Nelson *et al.* 2003; Raithatha *et al.* 2012). Finally, cyclin C-Cdk8p is required for COMPASS remodeling, which establishes a chromatin environment that permits RNA pol II recruitment, but not activation during cellular response to inositol deprivation. This remodeling event favors H3Lys4 2me in lieu of 3me and is a critical component to establish transcriptional memory to past stimuli (D’Urso *et al.* 2016). Our results indicate that cyclin C-Cdk8p may repress transcription in part by preventing the accumulation of histone H3Lys4 3me, consistent with this previously reported model.

We identified dramatic transcriptional upregulation of the *AQY1* aquaporin gene in *cnc1*Δ*jhd2*Δ yeast mutants cultured in nonfermentable carbon. Interestingly, *AQY1* overexpression confers yeast with enhanced freeze-thaw and peroxide stress tolerance (Tanghe *et al.* 2002; Linder *et al.* 2016). In addition, polymorphisms in this aquaporin are associated with resistance to multiple stresses and are selected for on the evolutionary scale (Will *et al.* 2010; Linder *et al.* 2016). The increased *AQY1* expression in *cnc1*Δ*jhd2*Δ yeast mutants is consistent with the previously identified roles for cyclin C in oxidative and metabolic stress responses. Specifically, yeast lacking *CNC1* are more resistant to oxidative stress, which may be due in part to elevated *AQY1* transcript levels in these mutants (Krasley *et al.* 2006). It is important to note that the *AQY1* allele present in the SK1 strain used in this study has been previously reported as nonfunctional, making it difficult to predict the impact of its increased transcription to stress tolerance (Will *et al.* 2010). However, since *AQY1* expression is induced during meiotic development and pseudohyphal formation, its constitutive activation in *cnc1*Δ*jhd2*Δ yeast mutants is consistent with the pseudohyphal phenotype observed in these mutants (Sidoux-Walter *et al.* 2004; Pettersson *et al.* 2005; Law and Ciccaglione 2015).

Why is *AQY1* so dramatically upregulated while other loci displaying increased H3Lys4 3me levels are not? Initial studies supported a model in which H3Lys4 3me is correlated with active transcription in multiple eukaryotes (Santos-Rosa *et al.* 2002; Schneider *et al.* 2004; Bernstein *et al.* 2005; Barski *et al.* 2007). More recent work has suggested that this modification mediates both transcriptional activation and, in some cases, repression (Venkatasubrahmanyam *et al.* 2007; Guillemette *et al.* 2011; Margaritis *et al.* 2012). Our results support a model in which H3Lys4 3me may provide a permissive environment for active transcription, but that this mark alone may be insufficient for inducing transcription (see below). This idea is supported by the concept that locus-specific transcriptional control is elicited by the combined activities of histone modifications and transcription factors (Rando and Winston 2012). In support of this, our ChIP-seq data found that H3Lys4 3me peaks specific to *cnc1*Δ*jhd2*Δ yeast mutants are enriched for Ste12p binding sites (Table S6). Ste12p, a transcriptional activator for pseudohyphal gene transcripts, is a kinase substrate for cyclin C-Cdk8p that becomes degraded following phosphorylation (Nelson *et al.* 2003). Interestingly, genome-wide ChIP-chip and ChIP-seq studies from independent laboratories have indicated that the *AQY1* promoter is bound directly by Ste12p as cells are grown in nutrient limited conditions that result in pseudohyphal induction (Borneman *et al.* 2006, 2007; Lefrancois *et al.* 2009; Zheng *et al.* 2010). Importantly, Ste12p promoter binding is a prerequisite for transcriptional induction of target genes (Zheng *et al.* 2010). Finally, yeast lacking *STE12* fail to activate *AQY1* transcription during nutrient deprivation, suggesting that direct interactions between Ste12p and the *AQY1* promoter are required for transcriptional induction during pseudohyphal differentiation (Madhani *et al.* 1999).

**Figure 6:**
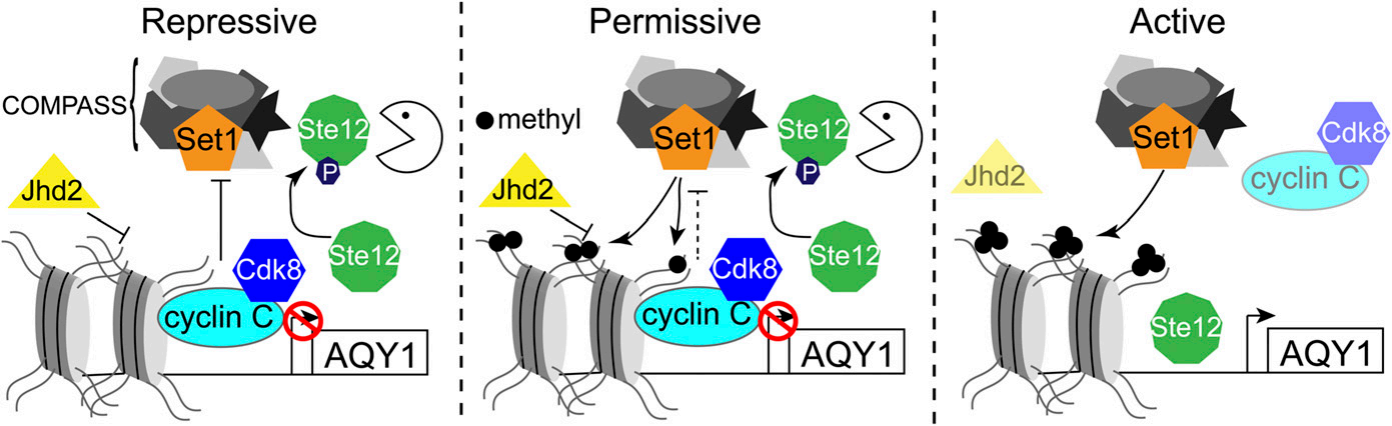
Putative stepwise model describing *AQY1* transcription. Transcriptional repression (left panel) of *AQY1* requires Jhd2p and cyclin C-Cdk8p activity. Jhd2p demethylates H3Lys4 while cyclin C-Cdk8p prevent Set1p/COMPASS binding and induce Ste12p proteolysis. Transcriptionally permissive chromatin (middle panel) is established by reduced cyclin C-Cdk8p activity, which allows Set1p/COMPASS to access the *AQY1* locus, but does not alleviate Ste12p proteolysis. H3Lys4 3me fails to accumulate in this condition due to Jhd2p-mediated demethylation. Active transcription (right panel) occurs when both cyclin C-Cdk8p and Jhd2p are inactivated. This allows H3Lys4 3me to accumulate and permits Ste12p stabilization and subsequent transcriptional induction. COMPASS, Complex of Proteins Associated with Set1; H3Lys4, histone 3Lys4.

Our results support a stepwise model in which both cyclin C-Cdk8p and Jhd2p regulate *AQY1* transcription. In this model, H3Lys4 methylation is inhibited by independent activities of Jhd2p, which demethylates H3Lys4, and cyclin C-Cdk8p, which prevents Set1p promoter binding, resulting in transcriptional repression (Figure 6, left panel). Transcriptionally permissive chromatin is established by reducing cyclin C-Cdk8p activity to allow Set1p recruitment, but H3Lys4 3me accumulation is prevented by Jhd2p (Figure 6, middle panel). Finally, transcriptional activation is achieved by eliminating cyclin C-Cdk8p and Jhd2p activity at the *AQY1* locus. This not only allows H3Lys4 3me accumulation, but also permits transcriptional activation by Ste12p, since it is no longer targeted for cyclin C-Cdk8p-mediated phosphorylation and proteolytic degradation (Figure 6, right panel). This multilayered transcriptional control mechanism confers cells with the ability to tune their transcriptional response in a manner that is appropriate with the stimulus. The presence of multiple factors that are required for transcription therefore provides a molecular buffer allowing cells to avoid extreme responses in the presence of moderate stressors.

It remains unclear whether cyclin C-Cdk8p-mediated H3Lys4 methylation inhibition acts via direct or indirect molecular mechanisms. Seminal work identified a role for *trans*-histone communication in which histone H2B ubiquitination is required for efficient H3Lys4 methylation (Krogan *et al.* 2003; Ng *et al.* 2003a,b). The Paf1 complex, which is thought to serve as a molecular platform for factors that regulate histone modifications and transcription, recruits the Rad6p and Bre1p components of the H2B ubiquitin machinery. In turn, the Paf1 complex subunit Rtf1p is required for H2B ubiquitination, Set1p recruitment, and efficient H3Lys4 methylation (Ng *et al.* 2003a,b). Our data are consistent with a model in which cyclin C-Cdk8p antagonize Rtf1p function to prevent Set1p recruitment and H3Lys4 methylation at target loci. Alternatively, cyclin C-Cdk8p may directly inhibit the COMPASS methyltransferase complex. In support of this, Brickner and colleagues recently proposed a model in which Cdk8p can directly prevent the association of the Spp1p subunit of the COMPASS complex during nutrient deprivation and transcriptional memory formation at the *INO1* promoter (D’Urso *et al.* 2016). While the authors demonstrated that Cdk8p is present at this locus as Spp1p is evicted, direct kinase targets responsible for this function remain elusive. Further mechanistic studies will be required to determine which of these models can explain how cyclin C-Cdk8p control H3Lys4 3me and transcription.

## Supplementary Material

Supplemental material is available online at www.g3journal.org/lookup/suppl/doi:10.1534/g3.117.039586/-/DC1.
