# Supplementary material for: The Saccharomyces cerevisiae Cdk8 Mediator Represses AQY1 Transcription by Inhibiting Set1p-Dependent Histone Methylation
Source: G3 (Bethesda). 2017 Jan 30;7(3):1001–10. doi: 10.1534/g3.117.039586 (PMC5345701; doi:10.1534/g3.117.039586)
Supplement: Supplementary file 1 [file 1001FigureS1.pdf]

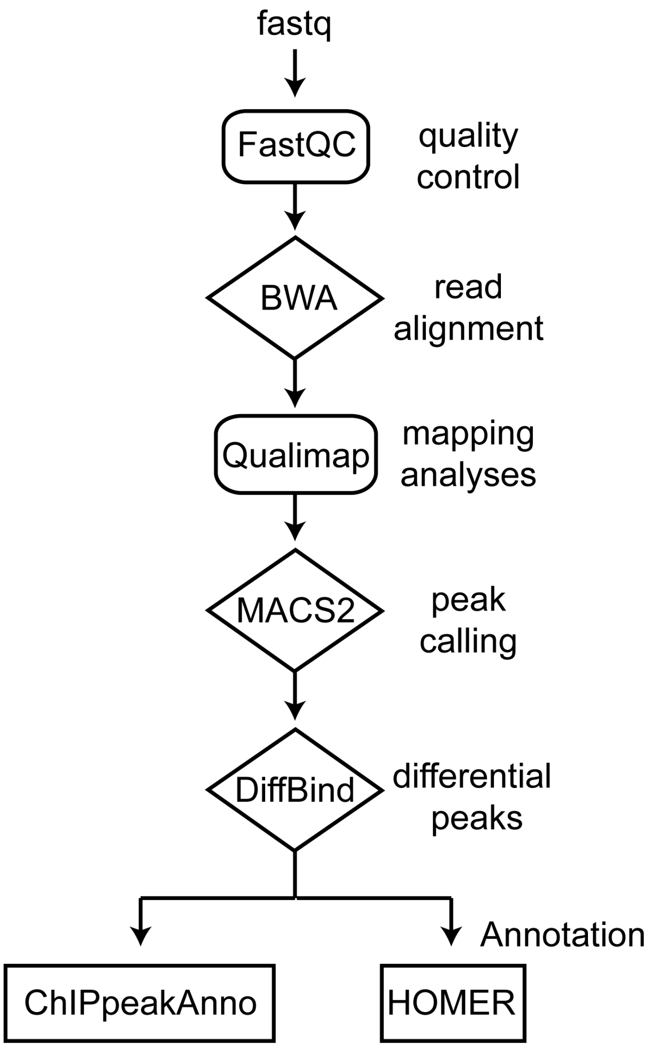

Figure S1 ChIP-seq computational pipeline. Following quality control, NGS sequence reads (.fastq files) were mapped to the SK1 genome using BWA. Differential peaks were called by invoking MACS2 and DiffBind, all peaks that were $\geq$ 2-fold enriched in *cnc1Δjhd2Δ* mutants were annotated. Resulting .bed files were first annotated by ChIPPeakAnno to identify the relationship of peak to neighboring ORF and the distribution of peaks relative to TSS; GO annotations and TF binding predictions were made by HOMER. See Materials and Methods section for more detail.
