# Supplementary material for: The Saccharomyces cerevisiae Cdk8 Mediator Represses AQY1 Transcription by Inhibiting Set1p-Dependent Histone Methylation
Source: G3 (Bethesda). 2017 Jan 30;7(3):1001–10. doi: 10.1534/g3.117.039586 (PMC5345701; doi:10.1534/g3.117.039586)
Supplement: Supplementary file 2 [file 1001FigureS2.pdf]

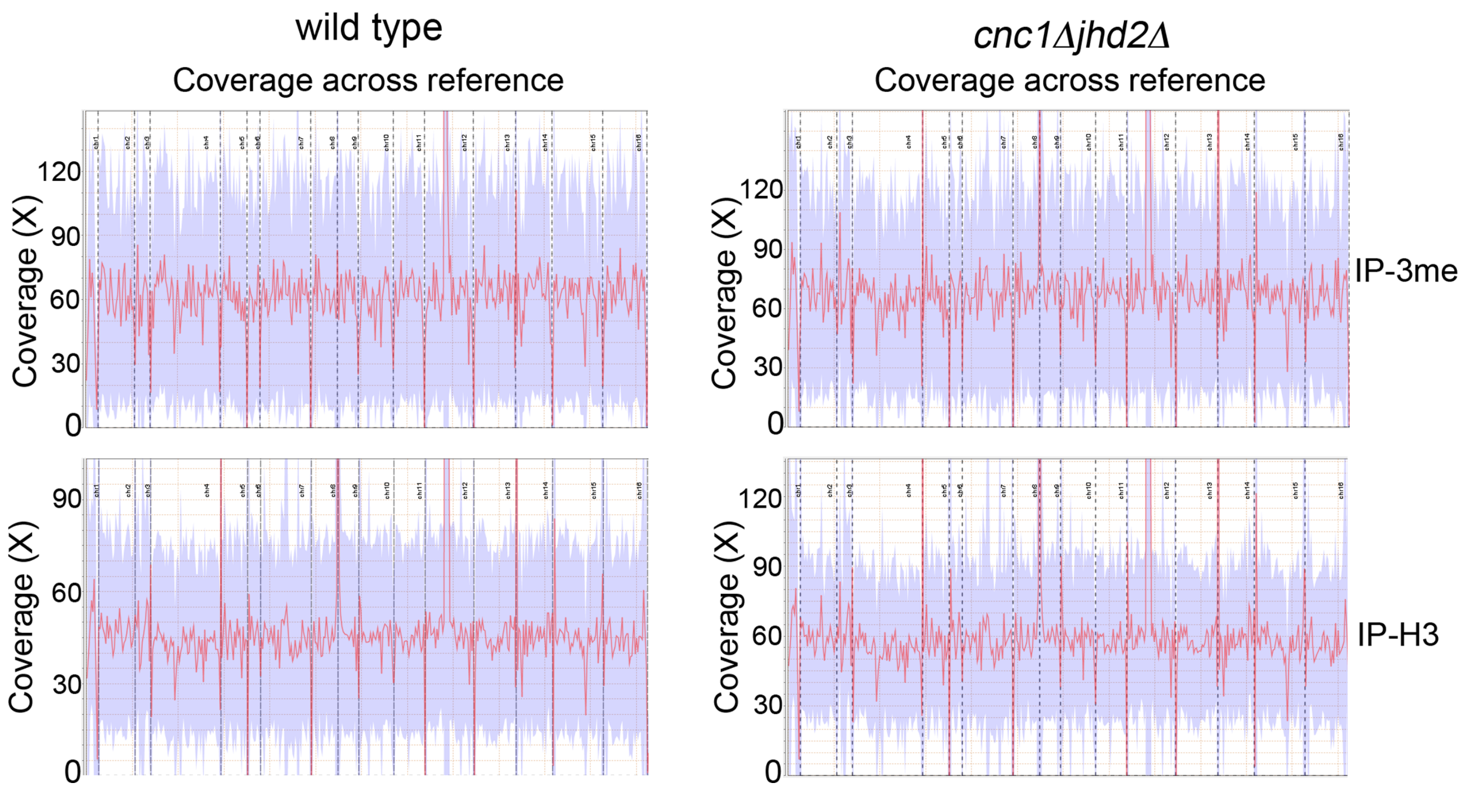


**Figure S2 Representative analyses of chromosomal coverage of mapped reads by Qualimap.** Fold coverage calculations performed by Qualimap for biological replicate 1, sequencing replicate 1 of ChIP-seq experiments. Red lines indicate the average fold coverage, shaded areas are the standard deviation fold coverage. Each chromosome is represented with vertical dashed lines separating them.
