# Supplementary material for: The Saccharomyces cerevisiae Cdk8 Mediator Represses AQY1 Transcription by Inhibiting Set1p-Dependent Histone Methylation
Source: G3 (Bethesda). 2017 Jan 30;7(3):1001–10. doi: 10.1534/g3.117.039586 (PMC5345701; doi:10.1534/g3.117.039586)
Supplement: Supplementary file 3 [file 1001FigureS3.pdf]

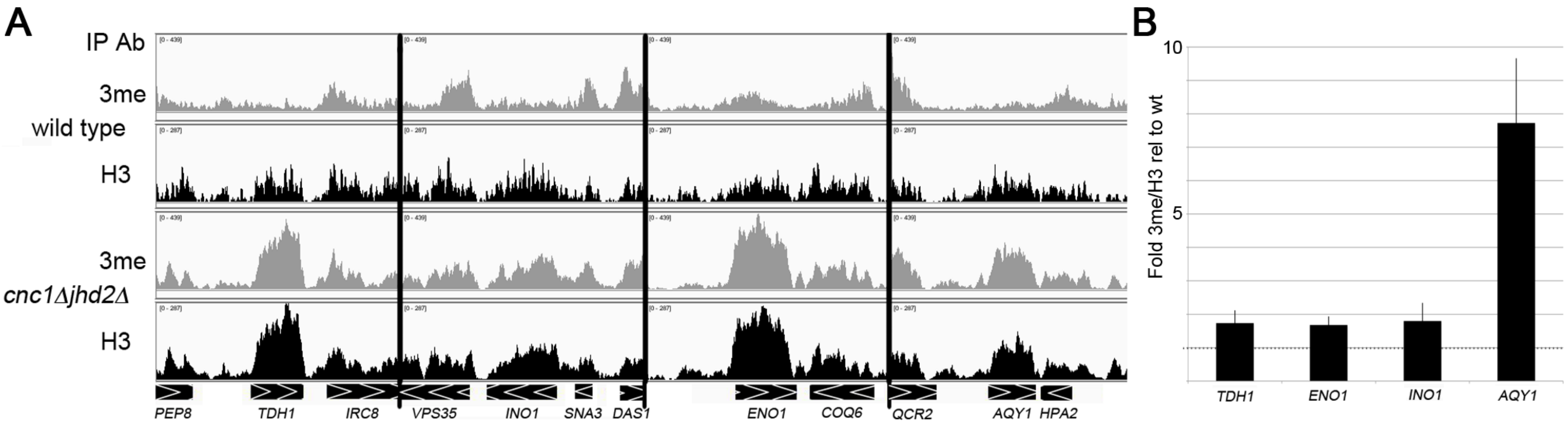

Figure S3 Reproducibility for differential H3Lys4 3me peaks. (A) Snapshot of the ChIP-seq mapped reads for H3Ly4 3me and H3 across the *TDH1*, *INO1*, *ENO1*, and *AQY1* loci that display elevated H3Lys4 3me/H3 signal in *cnc1Δjhd2Δ* mutants relative to wild type as determined by DiffBind. (B) ChIP-qPCR was performed on wild type and *cnc1Δjhd2Δ* mutants cultured to mid-logarithmic phase in non-fermentable carbon sources. Immunoprecipitations were performed as indicated; qPCR was directed towards top hits as identified by ChIP-seq. All values are normalized to wild type and represent the average of three independent biological replicates, error bars are standard deviations.
