# Supplementary material for: The Saccharomyces cerevisiae Cdk8 Mediator Represses AQY1 Transcription by Inhibiting Set1p-Dependent Histone Methylation
Source: G3 (Bethesda). 2017 Jan 30;7(3):1001–10. doi: 10.1534/g3.117.039586 (PMC5345701; doi:10.1534/g3.117.039586)
Supplement: Supplementary file 4 [file 1001FigureS4.pdf]

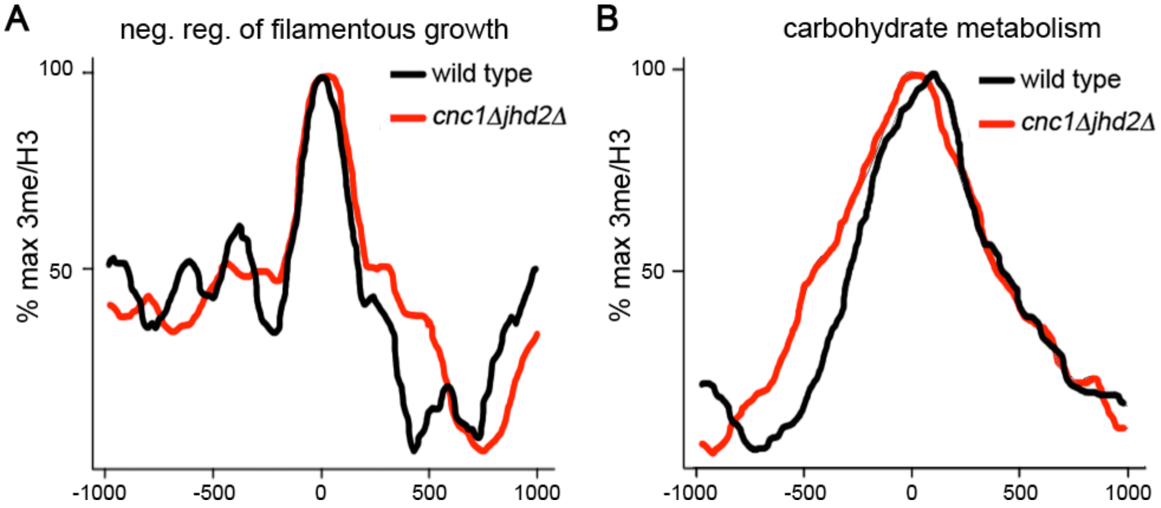

Figure S4 TSS curves for negative regulators of filamentous growth and genes involved in carbohydrate metabolism. H3Lys4 3me/histone H3 distribution was determined relative to the transcriptional start site of (A) negative regulators of filamentous growth or (B) carbohydrate metabolism genes.
