# Supplementary material for: The Saccharomyces cerevisiae Cdk8 Mediator Represses AQY1 Transcription by Inhibiting Set1p-Dependent Histone Methylation
Source: G3 (Bethesda). 2017 Jan 30;7(3):1001–10. doi: 10.1534/g3.117.039586 (PMC5345701; doi:10.1534/g3.117.039586)
Supplement: Supplementary file 5 [file 1001TableS1.pdf]

Table S1 qPCR primers used in this study

Primer name	Primer sequence (5’ -> 3’)
*AQY1*_I forward	TGTTTGCTTATGCGCGATGC
*AQY1*_I reverse	AATCGTGGTTTCGTCATGCC
*AQY1*_II forward	CCGTCGTTTATCCCTCAAAGC
*AQY1*_II reverse	TGGAAGGACATCAGGCATAAGG
*AQY1*_III forward	ACCGACAAGCAACATACACG
*AQY1*_III reverse	AGCGATGGTGCTTTGTTTCC
*AQY1*_IV forward	TGGAGAAGCGTGAAACCAAC
*AQY1*_IV reverse	AAGCGACGTGTGCGATAAAC
ENO1 forward	GCCGCTGCTGAAAAGAATGT
ENO1 reverse	TGGAGAGGTCTTGGACTTAGACAA
INO1 forward	TTTTGTTCCCGGCTTGGT T
INO1 reverse	CGTCTCCCGCAATGAATGTAC
TDH1 forward	CCACCGGTGTTTTCAAGGAA
TDH1 reverse	GGCACCAGCGTCAATGTG
ACT1 forward	TCGTTCCAATTTACGCTGGTT
ACT1 reverse	CGGCCAATCGATTCTCAA
